# Conservative Management of Cesarean Scar Pregnancy: A Case Report and Literature Review

**DOI:** 10.1155/2022/1793943

**Published:** 2022-06-22

**Authors:** Manuel Sánchez-Prieto, María Jesús Puy, Núria Barbany, Betlem Graupera, Maria Angela Pascual, Pere Barri-Soldevila

**Affiliations:** Department of Obstetrics and Gynecology, Instituto Universitario Dexeus, Barcelona, Spain

## Abstract

Cesarean scar pregnancy (CSP) is a rare form of ectopic pregnancy located in the lower uterine segment. The current increase in the percentage of cesarean sections is accompanied by significant growth in the incidence of CSP, while advances in ultrasound diagnostic techniques have led to a greater number of CSP diagnoses. A misdiagnosed CSP, or one that is diagnosed too late, is life-threatening to the pregnant patient and predisposes her to complications such as uterine bleeding or rupture, which often require hysterectomy and thus result in the irreversible loss of fertility. We present the case of a 50-year-old woman with a history of undiagnosed CSP after multiple consultations for intermittent bleeding and hemorrhage. She was diagnosed by ultrasound and the diagnosis was confirmed by hysteroscopy. She underwent conservative medical treatment that was successful.

## 1. Introduction

Cesarean scar pregnancy (CSP) is a type of ectopic pregnancy in which the gestational sac (GS) implants into the anterior wall of the lower uterine segment in an anterior cesarean scar [1]. It is the rarest form of ectopic pregnancy, representing 6% of all ectopic pregnancies in patients with a history of a previous cesarean section [1]. The incidence of CSP has increased, in parallel with the increase in the rate of cesarean sections and the widespread use of ultrasound in early pregnancy [2].

Abnormal implantation of the embryo within the myometrium and fibrous tissues of a previous scar can lead to uterine rupture, placental accreta, and uncontrolled bleeding, which can lead to hysterectomy and the permanent loss of fertility or even maternal death [3].

Early and accurate diagnosis and prompt management are very important to reduce life-threatening complications and preserve fertility [4]. Despite the large number of clinical reports and different therapeutic approaches, there is no consensus on the treatment of CSP [5].

Methotrexate (MTX) is an antimetabolite drug that has been used in the treatment of molar and ectopic pregnancies, including CSP [6, 7]. Although MTX is considered a safe and effective therapy, the optimal dose, route, and protocol for its use have yet to be determined for CSP [6].

We present a case of CSP detected in a symptomatic woman at the beginning of her pregnancy, and we review the management of the pathology.

## 2. Case report

A 50-year-old woman (Gravida 4, Para 1), with a history of 1 cesarean section and 3 rectovaginal fistula repairs -due to a complication of debridement of a Bartholin's gland abscess-, presented a menstrual delay. A urine pregnancy test was performed and was positive. Two days later, she started heavy bleeding. She went to the emergency department of an outer center, was diagnosed with an abortion in progress and underwent emergency dilation and curettage (D&C).

One month later, she consulted to another Center due to persistent bleeding and was diagnosed by ultrasound with chorionic remains in the left horn. A diagnostic hysteroscopy was recommended, but it was not performed since she expelled spontaneously. In the next 20 days, she had intermittent spotting.

**Figure 1:**
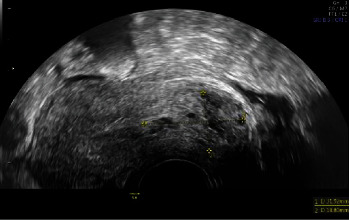
Transvaginal ultrasonographic study of the pelvic-abdomen region shows a retroverted uterus with a biometry of 70 mm x 40 mm. The uterine morphology was regular, and the myometrial ultrasound pattern was homogeneous. The endometrial thickness was 7 mm. The cervical canal was occupied by abundant blood content and clots with a size of 34 mm.

**Figure 2:**
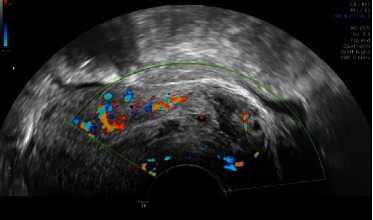
Color Doppler study did not provide new data for diagnosis.

On the day of her visit to our service, she came in due to vaginal bleeding with abundant clots. In the admission examination, abundant bleeding with clots at the level of the cervix was found, which were extracted with Foerster forceps. A transvaginal ultrasound was performed, which showed an endometrial thickness of 7 mm, with the cervical canal occupied by abundant blood content and clots with a size of 34 mm (Figures 1 and 2).

While waiting for ultrasound, she developed vasovagal syncope. Hypotension was evidenced, moderate bleeding continued with abundant clots, and the cervix was dilated to 1 cm.

Urgent analysis was performed, resulting in a hemoglobin level of 11.1 g/dL, correct coagulation, and a *β*-human chorionic gonadotropin (*β*-hCG) level of 2,000 mUI/mL.

D&C was performed by aspiration guided with transabdominal ultrasound, and 0.25 mg/ml of carboprost was administered intramuscularly. Correct hemostasis and no active bleeding were observed at the end of curettage.

Ultrasound and analytical control were recommended after one week.

The histological result of the curettage showed hematic material with fragments of decidua. Regarding analytical control, her *β*-hCG level had increased to 2188 mIU/mL.

**Figure 3:**
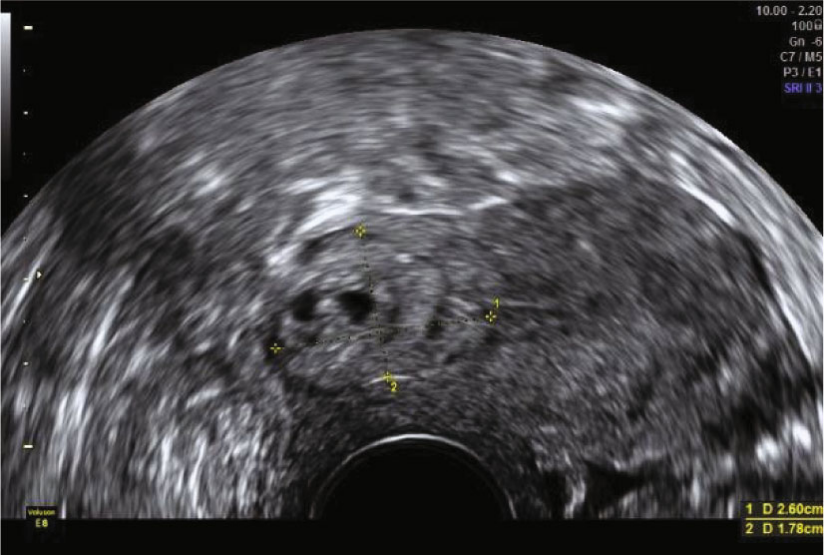
Transvaginal ultrasonographic study of the pelvic-abdominal region showing a retroverted uterus, with a biometry of 64 mm x 38 mm. The uterine morphology was regular, and the myometrial ultrasound pattern was homogeneous. Ultrasound signs of cavitary pathology were not observed. The endometrial thickness was 2 mm. At the level of the cesarean section scar, a 29x16 mm nodular image was observed, with a heterogeneous pattern.

**Figure 4:**
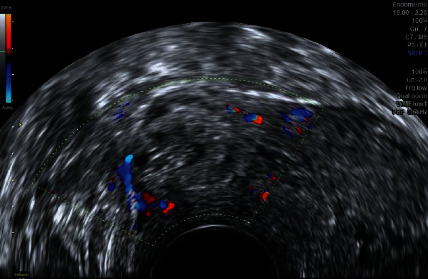
The color Doppler study showed vascularization.

Control transvaginal ultrasound revealed the following: At the level of the cesarean section scar, a 29 x 16 mm nodular image with a heterogeneous pattern was observed (Figure 3), and a color Doppler study showed vascularization (Figure 4). The image was compatible with organized and vascularized hematic material and compatible with probable placental remains, although it could not be conclusively ruled out that it had been an ectopic pregnancy.

**Figure 5:**
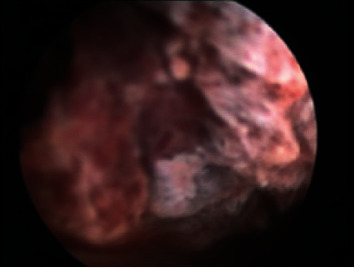
Hysteroscopic image showing an ectopic pregnancy at the level of the cesarean section scar.

Diagnostic hysteroscopy was recommended to confirm the suspected diagnosis; ectopic pregnancy was evidenced on the cesarean section scar (Figure 5).

**Figure 6:**
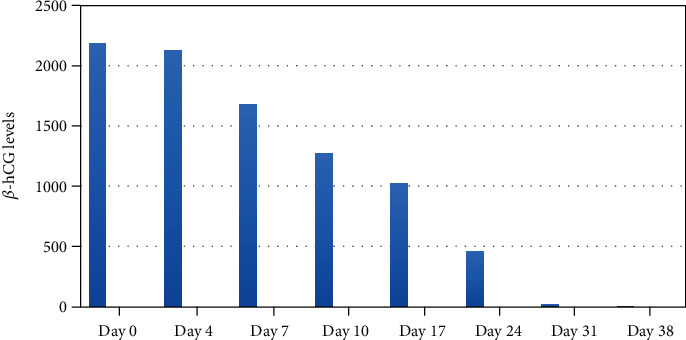
Chronogram of the serum *β*-hCG values.

The diagnosis of ovular remains in the previous cesarean section scar was confirmed, without being able to specify the gestation time. Given the diagnostic confirmation of ectopic pregnancy on the cesarean section scar, both surgical and medical treatment with methotrexate (MTX) was proposed so the patient could make an informed decision. She opted for conservative treatment. A single dose of MTX was administered. At 7 days, there was a >15% decrease in the *β*-hCG levels compared to the control on the 4th day, so no further doses were administered. On the 10th day, there was a >15% decrease compared to the 7th day, for which weekly clinical and analytical control was decided until *β*-hCG became negative. There was a gradual decrease in the *β*-hCG levels until negativization, 38 days after the administration of MTX (Figure 6).

**Figure 7:**
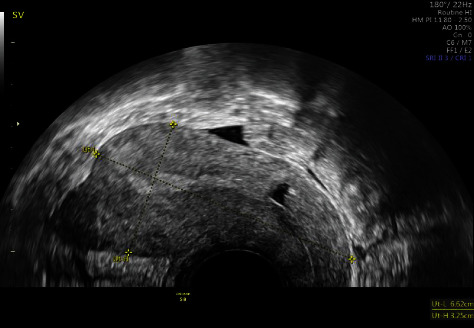
Transvaginal ultrasonographic study of the pelvic-abdominal region showed a retroverted uterus, with a biometry of 66 mm x 33 mm. Images of cavitary pathology were not observed. The endometrial thickness was 4 mm.

**Figure 8:**
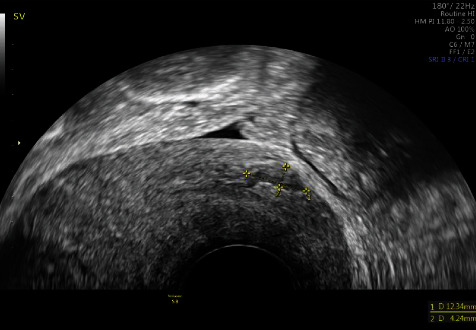
.12 mm x 4 mm niche.

Clinical and imaging controls were requested 2 months after MTX treatment. Ultrasound control was carried out, resulting in normality (Figure 7). A 12 x 4 mm niche was observed (Figure 8).

## 3. Discussion

Ectopic pregnancies in cesarean section scars (CSPs) are extremely rare, representing 0.4% of all pregnancies and constituting 6% of all ectopic pregnancies in patients with a history of a previous cesarean section [1]. They constitute a continuous pathology that ranges from gestation with implantation on a properly healed scar (superficial CSP) to those implanted in a dehiscent scar (“niche”) (deep CSP) which have a worse prognosis than those that are inserted on the scar [8].

The niche was defined as a cleft at the site of the cesarean section scar with a depth of at least 2 mm [9]. Basic measurements, including the niche length and depth, residual and adjacent myometrial thickness in the sagittal plane, and niche width in the transverse plane, were considered essential.

The ultrasound diagnostic criteria for CSP were defined as follows [4, 10, 11]:

1. Empty uterus with clearly visualized endometrium
2. Empty cervical canal
3. Gestational sac implanted in the lower anterior uterine segment at the presumed site of the cesarean section incision scar
4. Thin or absent myometrium between the gestational sac and the bladder (the majority of cases have a myometrium thickness<5 mm). “Sliding organ sign”
5. Doppler Flow at the previous cesarean scar

Although hysteroscopy and laparoscopy are not recommended diagnostic modalities, surgical findings at the time of surgical treatment are valuable in confirming the diagnosis of CSP [12].

### 3.1. Risk Factors

In a multivariate analysis, smoking in the first trimester (adjusted odds ratio (OR) 3.03, 95% CI 1.01-9.07), higher parity (adjusted OR 1.30, 95% CI 1.03-1.64) and more than 1 previous cesarean section (adjusted OR 3.43, 95% CI 1.35–8.66) were independently predictive of CSP [13]. An elective cesarean section in the index pregnancy was associated with an increased risk of CSP, but it did not remain significant in the multivariate analysis.

It is unknown whether the surgical technique of cesarean section affects the risk of a subsequent CSP [13].

### 3.2. Clinical Findings

In the early stages of pregnancy, most patients are asymptomatic. As the pregnancy progresses, vaginal bleeding can occur with or without pain [13]. The uterus can rupture and cause hemoperitoneum and hypovolemic shock.

In a review of 112 published cases and case series, the mean gestational age at presentation was 7.5 ± 2.5 weeks [13]. Among the 57 patients with presentation information, 37 percent were asymptomatic and diagnosed by ultrasound after remission for evaluation to rule out ectopic pregnancy, 39 percent had painless vaginal bleeding, 16 percent had abdominal pain and bleeding and 9 percent had only abdominal pain.

### 3.3. Risk Factors for Massive Bleeding

Multiple pregnancy, a big gestation sac, large gestation days, a high serum *β*-hCG level, an abundant blood supply to the pregnancy sac, and a thin myometrium may be risk factors for massive bleeding during CSP treatment [3].

### 3.4. Expectant Management

The meta-analysis by Calì et al. [14] showed that expectantly managed CSP with positive embryonic/fetal cardiac activity is associated with a high catastrophic maternal disease burden, including early uterine rupture, severe hemorrhage, abnormally invasive severe placenta, and hysterectomy.

Although the relative risk of maternal morbidity from the expectant management of CSP without cardiac activity is low, the risk of uterine rupture is as high as 13.4% [14]. The 2020 *Society for Maternal-Fetal Medicine* guideline also recommends against the expectant management of CSP (Grade 1B) [15].

### 3.5. Medical Treatment

Conservative treatment with systemic MTX is considered the treatment of choice in clinically stable patients with a desire to preserve fertility [1, 6, 7, 16].

In cases of positive embryocardium, as an alternative to the injection of 2 ml of KCl, the combined medical treatment with intrasaccular MTX + intramuscular MTX can be considered, since it is associated with an earlier negativization of *β*-HCG, the disappearance of the image of earlier ectopic gestation and a shorter hospitalization time [7].

### 3.6. Surgical Treatment

In some selected cases of CSP, the surgical option may be considered [1, 7, 16, 17]:

- Uterine curettage: Some authors [18] proposed performing aspiration curettage as the first therapeutic option in patients with superficial implantation who meet all the following diagnostic criteria: <8 weeks' gestation; a myometrial thickness between the bladder and gestational sac of >2 mm; and hemodynamic stability. In the case of suction curettage, it should be performed guided by transabdominal ultrasound, with a small cannula (4 or 6) and with a maximum suction pressure of 300 mmHg. It can be performed simultaneously with the help of a Foley balloon [18].
- Hysteroscopy: It is a therapeutic option with a low complication rate [19, 20] that can be considered as an alternative to curettage in patients with the same criteria described for uterine curettage
- Surgical resection by laparoscopy or laparotomy [17]: It is an option in cases in which there is bladder infiltration [20], as well as in cases of suspected uterine rupture
- Hysterectomy may be indicated in cases of uncontrollable bleeding or the impossibility of conservative treatment [7].

In cases of acute bleeding, selective embolization of the uterine arteries can be considered prior to any of the previously proposed therapeutic options [21, 22].

### 3.7. Outcomes of Reproduction after CSP

Women with a history of CSP still have a high pregnancy rate, but the risk of recurrence and miscarriage is also increased [23, 24]. The effect of different treatments on subsequent pregnancy is unclear [24].

There is a need to further evaluate whether cesarean section scar resection and repair can improve reproductive outcomes [24, 25].

## 4. Conclusion

Cesarean scar pregnancy (CSP) is a type of ectopic pregnancy in which the gestational sac (GS) implants into the anterior wall of the lower uterine segment in an anterior cesarean scar. Before a diagnosis of abortion in a woman with a history of uterine scarring, we must rule out the possibility of implantation in the scar. Knowledge of the specific ultrasound characteristics of the rare locations of ectopic pregnancies, such as CSP, is crucial to making a correct diagnosis and initiating prompt treatment to prevent complications and preserve a patient's fertility. Early pregnancy termination should be considered, and treatment options should be individualized.
